# A dual-center analysis of conservative versus liberal glycoprotein IIb–IIIa antagonist strategies in the treatment of ST-elevation myocardial infarction

**DOI:** 10.1038/s41598-024-64652-x

**Published:** 2024-07-01

**Authors:** Kashi Callichurn, Philippe Simard, Corrado De Marco, Payman Jamali, Yacine Saada, Alexis Matteau, Érick Schampaert, Samer Mansour, Raja Hatem, Brian J. Potter

**Affiliations:** 1 Faculty of Medicine, McGill University, Montreal, QC Canada; 2 Faculty of Medicine, Université de Montréal, Montreal, QC Canada; 3 Department of Medicine, Division of Cardiology, Centre Hospitalier de l’Université de Montréal (CHUM), Montreal, QC Canada; 4 CHUM Research Center (CRCHUM), Montreal, QC Canada; 5 Department of Medicine, Division of Cardiology, Hôpital du Sacré-Cœur de Montréal, Montreal, QC Canada; 6 Carrefour de l’innovation et Évaluation en Santé (CIÉS), Centre de Recherche du CHUM (CRCHUM), Cardiology and Interventional Cardiology, CHUM, Pavillon S, S03-334, 850, Rue St-Denis, Montréal, QC H2X 0A9 Canada

**Keywords:** Cardiology, Medical research

## Abstract

While the efficacy of GpIIb–IIIa-inhibitors during primary PCI (pPCI) for ST-elevated myocardial infarction (STEMI) has previously been demonstrated, its ongoing role and safety in combination with newer P2Y12-inhibitors is unclear. We therefore sought to compare outcomes between two centers with divergent approaches to the use of GpIIbIIIa antagonists in pPCI. We performed a retrospective chart review of all-comer STEMI patients treated with pPCI at two high-volume Montreal academic tertiary care centers. One center tended to use GpIIb–IIIa-inhibitors up-front in a large proportion of patients (liberal strategy) and the other preferring a bail-out approach (conservative strategy). Baseline patient characteristics and procedural data were compared between the two groups. The main efficacy outcome was rate of no-reflow/slow-reflow and the main safety outcome was BARC ≥ 2 bleeding events. A total of 459 patients were included, of whom 167 (36.5%) were exposed to a GpIIb–IIIa-antagonist. There was a significant overall difference in use of GpIIb–IIIa-antagonist between the two centers (60.5% vs. 16.1%, *p* < 0.01). Rate of no-reflow/slow-reflow was similar between groups (2.6% vs. 1.4%, *p* = 0.22). In-hospital rates of unplanned revascularization, stroke and death were also not different between groups. Use of a liberal GpIIb-–IIIa-antagonist strategy was however associated with a higher risk of bleeding (OR 3.16, 95% CI 1.57–6.37, *p* < 0.01), which persisted after adjustment for covariables (adjusted OR 2.85, 95% CI 1.40–5.81, *p* < 0.01). In this contemporary retrospective cohort, a conservative, bail-out only GpIIb-–IIIa-antagonist strategy was associated with a lower incidence of clinically relevant bleeding without any signal for an increase in no-reflow/slow-reflow or ischemic clinical events.

## Introduction

Ischemic complications of primary percutaneous coronary intervention (pPCI) represent a significant cause of procedure-related morbidity and mortality^1–3^. Among the range of possible ischemic complications, the so-called “no reflow” phenomenon of impaired distal perfusion despite addressing the culprit lesion and the risk of hyper-acute in-stent thrombosis remain chief concerns^1^, both of which are believed to be mediated by inadequate inhibition of platelet adhesion and aggregation^2,3^.

Platelet integrin glycoprotein IIb–IIIa (GpIIb–IIIa) antagonists interfere with fibrinogen binding, thereby inhibiting platelet aggregation, and may help prevent ischemic complications^4–6^. Several large, double-blinded, prospective studies have compared the use of GpIIb–IIIa antagonists to standard therapy with aspirin, heparin, and a thienopyridine in acute coronary syndromes or during percutaneous coronary intervention^5,6^. Collectively, these studies showed that the use of platelet GpIIb–IIIa receptor antagonists could prevent cardiac ischemic complications following percutaneous coronary intervention (PCI)^1^, especially in patients with ST-elevation myocardial infarction (STEMI)^2–6^, with reduced rates of myocardial infarction, death, and target vessel revascularization. However, the role and safety of GpIIb–IIIa antagonists in combination with more potent P2Y12 inhibitors, such as prasugrel and ticagrelor, is less clear, and the latest ACC/AHA/SCAI guidelines allow their use, but with a low level of evidence^7^. Indeed, there currently exists a paucity of data to support additional anti-ischemic and anti-thrombotic benefits of GpIIb–IIIa antagonists in patients receiving potent P2Y12 inhibitors, as well as a lack of data to support the safety in terms of bleeding complications of this antithrombotic combination. This lack of clear data in the literature translates clinically to a heterogenous practice with regards to GpIIb–IIIa administration in STEMI, with some operators continuing to use an upfront administration strategy and others preferring a more conservative strategy guided by the development of no-reflow/slow-reflow.

While more liberal use of GpIIb–IIIa-antagonists in the setting of potent P2Y12 inhibition may be associated with a reduction in ischemic complications, it may also increase the risk of bleeding. We therefore undertook a dual-center analysis to compare more conservative with more liberal use of the GpIIb–IIIa-antagonist eptifibatide in the setting of pPCI for STEMI.

## Methods

A retrospective analysis of all consecutive adult STEMI patients treated with pPCI and hospitalized at the PCI center for at least 24 h post-intervention from 2016 to 2019 was conducted at two high-volume Montreal academic tertiary care centers: The *Centre hospitalier de l’Université de Montréal* (CHUM) and *Hôpital du Sacré-Coeur de Montréal* (HSCM). As such, STEMI patients immediately returned to referring non-PCI hospitals were not eligible for inclusion in this study.

As the rate of GpIIb–IIIa antagonist use was known to be higher at HSCM, with a greater proportion benefiting from up-front administration (as opposed to a bail-out strategy for persistent thrombosis or no-reflow), the hospital of admission was our primary unit of analysis with HSCM representing a more liberal GpIIb–IIIa-inhibitor use strategy and the CHUM representing a more conservative one. Both HSCM and the CHUM are teaching hospitals affiliated with the *Université de Montréal* Faculty of Medicine and have otherwise very similar STEMI care pathways. Antithrombotic management at the time of pPCI was at the operator’s discretion. Eptifibatide is the only GpIIb–IIIa antagonist used in both centers.

Clinical characteristics, including demographic data, past medical history, and the details of the index STEMI hospitalization were extracted from a combination of patients’ hospital medical records and catheterization laboratory databases.

The primary efficacy outcome was the rate of no-flow/slow-flow during the pPCI procedure defined as a TIMI flow score less than 3 after initial treatment of the culprit lesion. Secondary ischemic outcomes included in-hospital urgent revascularization, stent thrombosis, myocardial infarction, stroke and death. The primary safety outcome was in-hospital major bleeding, defined as Bleeding Academic Research Consortium (BARC) types 2–5 bleeding events. BARC 3–5 bleeding events were also recorded as a secondary safety outcome.

Normally distributed continuous variables are described as means ± standard-deviations or, otherwise, as medians with associated interquartile ranges for non-normally distributed variables. Discrete variables are reported as frequencies and percent proportions. The primary comparison of interest was between conservative (CHUM) and liberal (HSCM) GpIIb–IIIa-antagonist strategies (intention-to-treat analysis). In secondary analyses, patients treated with a GpIIb–IIIa antagonist were compared to those who were not irrespective of the center of admission (on-treatment analysis). Variables were compared between groups using the student t-test, Wilcoxon rank sum test, or chi-square test, as appropriate. Logistic regression analysis was planned to analyze the effect of GpIIb–IIIa-antagonist strategy (intention-to-treat analysis) and treatment (on-treatment analysis) on both efficacy and safety outcomes, provided that the unadjusted results were significant, with variable selection based on the results of univariate analyses and expert opinion. A *p*-value of < 0.05 was considered statistically significant for all analyses in keeping with the exploratory nature of this study. Statistical analyses were performed using SPSS, version 25 (SPSS Inc., Chicago IL) and the study was approved by the institutional review boards of both centers (*comités d’éthique en recherche* CHUM and HSCM) with a waiver for informed consent. This study was performed in accordance with the Declaration of Helsinki.

## Results

A total of 459 patients undergoing pPCI for STEMI (mean age 63.9 ± 12.3 years, 76.7% male) met our inclusion criteria. Baseline clinical characteristics are presented in Table 1 and were overall similar between centers. However, there was a higher prevalence of liver disease (0% vs. 2.8%, *p* = 0.01), labile INR (0% vs. 2.0%, *p* = 0.04) and history of previous clinically significant bleeding (4.8% vs. 10.0%, *p* = 0.03) at the conservative strategy center, while baseline use of NSAIDs and/or antiplatelet drugs was higher at the liberal strategy center (24.3% vs. 11.2%, *p* < 0.01), as with rivaroxaban use (3.8% vs. 0.8%, *p* = 0.003).

**Table 1:** Baseline clinical characteristics.

Characteristic	All	Up-front strategy	Bailout strategy	*P*-value
Age	63.9 ± 12.3	64.2 ± 12.4	63.7 ± 12.2	0.611
Male	352 (76.7)	164 (78.1)	188 (75.5)	0.513
Length of stay (days)	5.02 ± 10.0	4.44 ± 5.11	5.51 ± 12.76	0.254
Use of GpIIbIIIa	167 (36.4)	127 (60.5)	40 (16.1)	< 0.001
Diabetes mellitus (DM)	104 (22.7)	55 (26.2)	49 (19.7)	0.097
Hypertension (HTN)	231 (50.3)	105 (50.0)	126 (50.6	0.898
Uncontrolled HTN at admission (SBP > 160 mmHg)	14 (3.1)	10 (4.8)	4 (1.6)	0.05
Stroke	33 (7.2)	15 (7.1)	18 (7.2)	0.972
Vascular disease	79 (17.2)	31 (14.8)	48(19.3)	0.202
Heart failure	17 (3.7)	10 (4.8)	7 (2.8)	0.27
Chronic kidney disease (CKD)	33 (7.5)	16 (7.6)	17 (6.8)	0.744
Chronic liver disease	7 (1.5)	0	7 (2.8)	0.014
History of previous major bleeding	35 (7.6)	10 (4.8)	25 (10.0)	0.034
History of labile INR	5 (1.1)	0	5 (2.0)	0.039
Drugs associated with increased risk of bleeding*	79 (17.2)	51 (24.3)	28 (11.2)	< 0.001
Alcoholism	46 (10.0)	19 (9.1)	27 (10.8)	0.534
Height (m)	1.71 ± 0.09	1.71 ± 0.10	1.71 ± 0.09	0.538
Weight (kg)	79.2 ± 18.0	80.9 ± 20.3	77.9 ± 15.9	0.102
BMI (kg/m2)	26.8 ± 6.3	26.7 ± 4.7	25.6 ± 7.4	0.06
Creatinine (mmol/L)	89.1 ± 42.5	89.1 ± 49.4	89.1 ± 35.7	0.987

Procedural data are presented in Table 2. There was a significantly higher proportion of patients presenting in cardiogenic shock at the liberal strategy center (21.0% vs. 10.8%, *p* < 0.01). There was a significantly higher use of ticagrelor (68.1% vs. 53.4%, *p* < 0.01) at the liberal center, but more use of prasugrel at the conservative center (28.9% vs. 18.1%, *p* < 0.01). Initial pre-procedural TIMI flow was statistically higher in the liberal center (*p* = 0.04) with a higher proportion of TIMI flow 2 and 3. Overall, 36.4% of patients were treated with GpIIb–IIIa-inhibitors. As expected, the rate of GpIIb–IIIa antagonist use was higher at HSCM (60.5%) than at the CHUM (16.1%), with an increased use of both GpIIb–IIIa boluses (59.5% vs. 15.7%, *p* < 0.001) and perfusions (57.6% vs. 7.6%, *p* < 0.001) at the liberal center.

**Table 2:** Procedural data.

Characteristic	All	Up-front strategy	Bailout strategy	*P*-value
Acute coronary syndrome (ACS)	451 (98.3)	202 (96.2)	249 (100)	0.002
Shock	71 (15.5)	44 (21.0)	27 (10.8)	0.003
Cardiac Arrest	57 (12.4)	24 (11.4)	33 (13.3)	0.555
Procedural anticoagulation				
Unfractionated heparin (UFH)	432 (94.1)	203 (96.7)	229 (92.0)	0.001
Bivalirudin	18 (3.9	1 (0.5)	17 (6.8)	
Other	6 (1.3)	5 (2.4)	1 (0.4)	
TIMI flow score pre-intervention – mean (interquartile)	0	0 (0;1)	0 (0;0)	
0	333 (76.2)	140 (71.8)	193 (79.8)	0.222
1	22 (5.0)	10 (5.1)	12 (5.0)	
2	31 (7.1)	17 (8.7)	14 (5.8)	
3	51 (11.7)	28 (14.4)	23 (9.5)	
Femoral access	61 (13.3)	27 (12.9%)	34 (13.7%)	0.538
Use of bare metal stent (BMS)	3 (0.7)	1 (0.5)	2 (0.8)	0.665
Use of drug-eluting stent (DES)	442 (96.3)	201 (95.7)	241 (96.8)	0.544
Use of 2nd generation DES	440 (99.3)	199 (99.0)	241 (99.6)	0.198
Median number of lesions (Interquartile)	1 (1;2)	1 (1;2)	1 (1;2)	
Mean number of lesions	1.66 ± 0.93	1.56 ± 0.85	1.74 ± 0.99	0.035
0	4 (0.9)	2 (1.0)	2 (0.8)	
1	252 (54.9)	124 (59.0)	128 (51.4)	
2	124 (27.0)	57 (27.1)	67 (26.9)	
3	63 (13.7)	20 (9.5)	43 (17.3)	
4	10 (2.2)	5 (2.4)	5 (2.0)	
5	4 (0.9)	2 (1.0)	2 (0.8)	
6	1 (0.2)	0	1 (0.4)	
7	1 (0.2)	0	1 (0.4)	
Median number of stents	1 (1;2)	1 (1;2)	1 (1;2)	
Mean number of Stents	1.70 (1.01)	1.7 ± 0.9	1.7 ± 1.1	0.986
0	15 (3.3)	8 (3.8)	7 (2.8)	
1	222 (48.5)	98 (46.7)	124 (50.0)	
2	143 (31.2)	67 (31.9)	76 (30.6)	
3	52 (11.4)	24 (11.4)	28 (11.3)	
4	21 (4.6)	12 (5.7)	9 (3.6)	
5	2 (0.4)	1 (0.5)	1 (0.4)	
6	0	0	0	
7	3 (0.7)	0	3 (1.2)	
Left main (LM) stent	14 (3.1)	8 (3.8)	6 (2.4)	0.385
Left anterior descending (LAD) stent	223 (48.6)	97 (46.2)	126 (50.6)	0.346
Circumflex (Cx) stent	74 (16.1)	31 (14.8)	43 (17.3)	0.467
Right coronary artery (RCA) stent	179 (39.0)	93 (44.3)	86 (34.5)	0.033
Coronary bypass stent	8 (1.7)	2 (1.0)	6 (2.4)	0.235
TIMI flow post procedure—median (interquartile)	3	3 (3;3)	3 (3;3)	
0	8 (1.8)	4 (2.1)	4 (1.6)	
1	1 (0.2)	1 (0.5)	0	
2	16 (3.6)	5 (2.6)	11 (4.4)	
3	415 (94.3)	182 (94.8)	233 (94.0)	
Timing from chest pain to access	162.3 ± 191.3	108.7 ± 156.8	200.9 ± 204.4	< 0.001
GpIIbIIIa bolus during procedure	164 (35.7)	125 (59.5)	39 (15.7)	< 0.001
GpIIbIIIa perfusion during procedure	140 (30.5)	121 (57.6)	19 (7.6)	< 0.001
Thrombo-aspiration	256 (55.8)	110 (52.4)	146 (58.6)	0.179
Use of adenosine or nipride	44 (9.6)	14 (6.7)	30 (12.0)	0.051

Efficacy and safety outcomes are shown in Table 3**.** The rate of no reflow/slow reflow was similar in both groups (2.6% vs. 1.6%, *p* = 0.47; OR: 1.63, 95% CI 0.43–8.16). Both cohorts were comparable in terms of in-hospital urgent revascularization, stent thrombosis, stroke, cardiovascular mortality, or all-cause mortality. However, there was a higher proportion of in-hospital MI at the liberal center (3.8% vs. 0.4%, *p* = 0.01).

**Table 3:** Efficacy and safety outcomes.

	All	Up-front strategy	Bailout strategy	*P*-value
Bleeding BARC ≥ 3	17 (3.7)	10 (4.8)	7 (2.8)	0.27
Bleeding BARC ≥ 2	41 (8.9)	29 (13.8)	12 (4.8)	0.001
No bleeding (BARC 0)	408 (88.9)	171 (81.4)	237 (95.2)	
Bleeding BARC 1	10 (2.2)	10 (4.8)	0	
Bleeding BARC 2	24 (5.2)	19 (9.0)	5 (2.0)	
Bleeding BARC 3	17 (3.7)	10 (4.8)	7 (2.8)	
Unplanned revascularization	9 (2.0)	5 (2.4)	4 (1.6)	0.551
New ST-elevation following intervention	5 (1.1)	4 (1.9)	1 (0.4)	0.122
New myocardial infarction	9 (2.0)	8 (3.8)	1 (0.4)	0.009
Death (any cause)	19 (4.1)	8 (3.8)	11 (4.4)	0.745
Death (cardiovascular cause)	2 (0.4)	1 (0.5)	1 (0.4)	0.904
Death (unknown cause)	17 (3.7)	7 (3.3)	10 (4.0)	0.7
Stroke	6 (1.3)	3 (1.4)	3 (1.2)	0.833

The primary safety outcome event, in-hospital BARC ≥ 2 bleeding events, was more frequently observed with a liberal GpIIb–IIIa-antagonist strategy (unadjusted OR: 3.16, 95% CI 1.57–6.37, *p* < 0.01). When adjusted for covariables in the multivariate logistical regression model (Supplemental Table S3A), the risk of bleeding remained higher in the liberal strategy group (adjusted OR: 2.85, 95% CI 1.40–5.81, *p* < 0.01). However, BARC ≥ 3 bleeding events were comparable between groups.

In the on-treatment analysis, there was no significant difference in the rate of no reflow/slow reflow between patients exposed to GpIIb–IIIa inhibitors and those not exposed (3.1% vs. 1.4%, *p* = 0.22). Use of GpIIb–IIIa was associated with a greater risk of BARC ≥ 3 and BARC ≥ 2 bleeding events (OR: 4.44, 95% CI 1.54–12.85, *p* < 0.01; OR: 3.41, 95% CI 1.75–6.64, *p* < 0.01, respectively).

## Discussion

In this retrospective dual-center study, we found that a more liberal GpIIb–IIIa antagonist strategy in combination with contemporary potent dual antiplatelet therapy in the context of pPCI for STEMI was not associated with any improvement in ischemic outcomes compared to a more conservative strategy but was associated with more clinically significant bleeding.

Atherothrombotic events occur when vascular endothelial injury causes platelet activation and aggregation leading to thrombosis, ischemia, and ultimately infarction. In order for platelet aggregation to occur, the co-activation of two platelets receptors, P2Y_1_ and P2Y_12_, is necessary for generating adenosine diphosphate (ADP)^8^, with the P2Y_12_ receptor being the predominant receptor responsible for ADP. Increased ADP leads to activation of the GpIIb–IIIa receptor, causing platelet degranulation and amplification of platelet aggregation^9^. Adjunctive medical management of STEMI patients is therefore focused on preventing post-ACS and, more specifically, post-pPCI complications through inhibition of platelet aggregation^10,11^. The mechanism underlying the benefits of GpIIb–IIIa inhibition results, ultimately, from decreased incretin $\alpha$_IIb_$\beta$_3_, which plays a central role in platelet activation, aggregation, and adhesion^12–15^ and whose upregulation has been linked with adverse cardiovascular events in patients with genetic polymorphisms^14^.

Multiple studies have shown the benefits of GpIIb–IIIa receptor antagonist use in addition to ASA monotherapy or clopidogrel-based dual antiplatelet therapy (DAPT) in preventing cardiac ischemic complications^5,7,16,17^, notably in terms of reducing the rates of myocardial infarction, death, and re-intervention for revascularization of the culprit vessel following PCI^18^. However study published in 2021^19^ showed that cangrelor, a potent intravenous continuous infusion P2Y12 inhibitor, was a safe and effective alternative to GpIIb–IIIa antagonism, suggesting that GpIIb–IIIa inhibitors might have limited benefit in the setting of potent P2Y12 inhibition. The global availability of cangrelor is variable, which limits the applicability of these findings, and it it remains unclear whether these findings would extend to the newer oral P2Y12 inhibitors ticagrelor and prasugrel. Additionally, any anti-thrombotic benefits of GpIIb–IIIa antagonists are likely to be off-set by an increased risk of bleeding and thrombocytopenia^20^. The role and safety of combining GpIIb–IIIa antagonists with the more potent oral P2Y12 inhibitors is therefore unknown.

A sub-group analysis of the POPular Genetics^21^ trial, comparing GpIIb–IIIa to oral antiplatelet therapy alone, showed that GpIIb–IIIa receptor antagonist use was associated with fewer thrombotic events and early myocardial infarctions. The FASTER multi-center registry similarly showed a lower incidence of major adverse cardiovascular events in patients receiving GpIIb–IIIa antagonists during pPCI^22^. However, a sub-group analysis of the ATLANTIC^23^ trial that included STEMI patients treated with ticagrelor-DAPT, found no difference in 30-day ischemic outcomes between groups receiving and not receiving GpIIb–IIIa antagonists. Our findings add significantly to the literature, in both confirming the results of ATLANTIC and extending the evidence base to patients treated with prasugrel, as our population included a higher proportion of patients receiving prasugrel than previously reported.

We observed a similar rate of ischemic complications in both groups. Indeed, patients at HSCM who were treated with a more liberal GpIIb–IIIa strategy did not have a lower rate of post-procedural ischemic complications, and even showed a tendency towards an increased rate of post-procedural MI. One possible explanation for this seemingly paradoxical effect is the interruption of anticoagulation or antiplatelet therapy in the setting of a bleeding complication that could have predisposed patients to a higher risk of post-procedure MI. However, patients treated with GpIIb–IIIa antagonists at HSCM had a higher prevalence of cardiogenic shock, which could have also predisposed these patients to a higher risk of early thrombotic events^24^.

The observed increase in clinically relevant bleeding events is consistent with multiple studies having shown that combination antiplatelet therapies result in incremental bleeding risk over less intensive platelet inhibition^17,21^. The lack of a significant difference in major bleeding (BARC 3–5) may be explained by a lack of rigorous prospective assessment of this outcome or the relatively low overall frequency of this outcome in our cohort. Indeed, we found lower rates of major bleeding compared to previous studies, but the literature is also divided on this issue. In the sub-group analysis of the ATLANTIC trial, administration of GpIIb–IIIa antagonists was associated with a significant increase in 30-day non-CABG related PLATO major bleeding^23^. However, the FASTER registry, in contrast, showed a low incidence of major bleeding in patients receiving a GpIIb–IIIa receptor antagonist^22^. The lower rates of major bleeding seen in our study could be attributed to the fact that both study centers are high-volume radial access centres, an approach associated with fewer bleeding complications. As such, one might expect the rate of bleeding events to be higher in centers using primarily femoral access for STEMI patients. Indeed, most bleeding events in our study, regardless of GpIIb–IIIa strategy, were upper gastro-intestinal or oropharyngeal (i.e., non-access site-related). Bleeding events were associated with vitamin K antagonist use at baseline, direct oral anticoagulant use, uncontrolled hypertension, heart failure, cardiogenic shock, and kidney disease. The effect of GpIIb–IIIa strategy on bleeding events remained significant after controlling for different combinations of these factors (Supplemental Table S3).

## Limitations

Our study’s primary limitations relate to its retrospective nature, which limits our ability to establish causation between the strategy of GpIIb–IIIa receptor antagonist use and observed clinical outcomes. Additionally, despite adjustment for baseline characteristics associated with bleeding, because of a limited number of clinical events, residual confounding is a possibility. Additionally, the studied outcomes were not prospectively ascertained or validated. Additionally, the sample size and observed event rates, even if they had been prospectively obtained, are likely insufficient to make definitive conclusions regarding the role of GpIIb–IIIa antagonists in the setting of contemporary dual antiplatelet therapy. Further study is therefore required. Moreover, the GpIIb–IIIa antagonist strategies studied were not protocolized or algorithm based, but at the discretion of the interventional cardiologist, which raises the specter of selection bias. While the rates of GpIIb–IIIa use were sufficiently divergent between centers that it is unlikely that differential baseline characteristics and selection bias can explain the start difference in GpIIb–IIIa antagonist use, such selection bias and confounding may have skewed the results towards neutrality. Only a prospective randomized study can truly answer this clinical question. but whether such a study would be broadly clinically acceptable given our findings remains a matter of debate. Larger collaborative registries may also yield satisfactory data to guide clinical practice. Additional unanswered questions that could be addressed through prospective studies include the optimal timing and dose of GpIIb–IIIa antagonist administration in the context of more potent P2Y12 inhibitors.

## Conclusion

In this contemporary STEMI cohort, a more liberal strategy of GpIIb–IIIa-antagonist use during pPCI, including a higher rate of “up-front” GpIIb–IIIa-antagonist loading, was not associated with a reduction in early ischemic outcomes compared to a more conservative strategy. Moreover, the liberal strategy was associated with a higher risk of bleeding in a population treated with contemporary potent oral dual antiplatelet therapy. A more conservative approach may therefore be preferred. Larger, prospective studies are warranted to better define the role of GpIIb–IIIa antagonists in the modern era.

### Supplementary Information

Supplementary Tables.

## Data Availability

The datasets used and/or analyzed during the current study are available from the corresponding author on reasonable request.
