# Supplementary material for: A dual-center analysis of conservative versus liberal glycoprotein IIb–IIIa antagonist strategies in the treatment of ST-elevation myocardial infarction
Source: Sci Rep. 2024 Jul 1;14:15003. doi: 10.1038/s41598-024-64652-x (PMC11217494; doi:10.1038/s41598-024-64652-x)
Supplement: Supplementary file 1 — Supplementary Tables. [file 41598_2024_64652_MOESM1_ESM.docx]

**Supplemental Appendix**

**A Dual-Center Analysis of Conservative vs. Liberal Glycoprotein IIb-IIIa Antagonist Strategies in the Treatment of ST-Elevation Myocardial Infarction**

Kashi Callichurn MD,^1*^ Philippe Simard MD,^2*^ Corrado De Marco MD,^2^

Payman Jamali MD,^2^ Yacine Saada MD,^2^ Alexis Matteau MD MSc,^1,3,4^

Érick Schampaert MD,^5^ Samer Mansour MD,^1,3,4^ Raja Hatem MD,^5^

& Brian J. Potter MDCM SM. ^1,3,4^

*Co-First Authors with equal contribution

1. Faculty of Medicine, McGill University, Montreal, QC.
2. Faculty of Medicine, Université de Montréal, Montreal, QC.
3. Department of Medicine, Division of Cardiology, Centre hospitalier de l’Université de Montréal (CHUM), Montreal, QC.
4. CHUM Research Center (CRCHUM), Montreal, QC.
5. Department of Medicine, Division of Cardiology, Hôpital du Sacré-Cœur de Montréal, Montreal, QC.

Short Title: Comparison of GpIIb-IIIa Antagonist Strategies

Brief Summary: 67 words

Abstract: 247 words

Text & References: 2618 words

Total: 3414 words

Author of correspondence:

Brian J. Potter, MDCM SM FRCPC

Carrefour de l’innovation et évaluation en santé (CIÉS),

Centre de recherche du CHUM (CRCHUM)

Cardiology & Interventional Cardiology, CHUM

Pavillon S, S03-334

850, rue St-Denis

Montréal, QC, Canada

H2X 0A9

[brian.potter@umontreal.ca](mailto:brian.potter@umontreal.ca)

Tel: 514-890-8000 ext.15473

## Fax: 514-412-7212

## **Table S1. Safety and efficacy outcomes according GpIIbIIIa strategy (conservative as reference).**

| **Outcome** | **B** | **SE** | **Wald** | **p value** | **Exp B (odds ratio)** | **CI** |
| --- | --- | --- | --- | --- | --- | --- |
| **Bleeding BARC ≥ 3** | 0.547 | 0.502 | 1.189 | 0.276 | 1.729 | 0.646 - 4.624 |
| **Bleeding BARC ≥ 2** | 1.152 | 0.357 | 10.403 | 0.001 | 3.164 | 1.571 - 6.372 |
| **Unplanned revascularization** | 0.401 | 0.677 | 0.351 | 0.554 | 1.494 | 0.396 - 5.636 |
| **New ST-elevation** | 1.572 | 1.122 | 1.963 | 0.161 | 4.816 | 0.534 - 43.420 |
| **New myocardial infarction** | 2.285 | 1.065 | 4.603 | 0.032 | 9.822 | 1.218 - 79.181 |
| **Death (any cause)** | -0.154 | 0.474 | 0.106 | 0.745 | 0.857 | 0.338 - 2.171 |
| **Death (cardiovascular cause)** | -0.193 | 0.502 | 0.148 | 0.7 | 0.824 | 0.308 - 2.204 |
| **Death (unknown cause)** | 0.173 | 0.822 | 0.044 | 0.834 | 1.188 | 0.237 - 5.951 |

## **Table S2. Univariate logistic regression models for risk of bleeding BARC ≥ 2**

| **Candidate variable** | **B** | **SE** | **Wald** | **p value** | **Exp B**  **(odds ratio)** | **CI** |
| --- | --- | --- | --- | --- | --- | --- |
| **Baseline Characteristics** | | | | | | |
| **Age** | 0.034 | 0.013 | 6.76 | 0.009 | 1.035 | 1.008 - 1.062 |
| **Male** | -0.341 | 0.363 | 0.887 | 0.346 | 0.711 | 0.349 - 1.447 |
| **Length of stay** | 0.03 | 0.016 | 3.634 | 0.057 | 1.031 | 0.999 - 1.063 |
| **G2B3A** | 1.225 | 0.34 | 12.962 | <0.001 | 3.405 | 1.748 - 6.635 |
| **DM** | 0.383 | 0.363 | 1.112 | 0.292 | 1.466 | 0.720 - 2.987 |
| **HTN** | 0.475 | 0.335 | 2.013 | 0.156 | 1.608 | 0.834 - 3.099 |
| **Uncontrolled HTN at admission** | 1.842 | 0.584 | 9.945 | 0.002 | 6.312 | 2.008 - 19.836 |
| **Stroke** | 0.021 | 0.629 | 0.001 | 0.974 | 1.021 | 0.298 - 3.503 |
| **Vascular disease** | -0.11 | 0.435 | 0.001 | 0.98 | 0.989 | 0.422 - 2.320 |
| **HF** | 1.214 | 0.597 | 4.137 | 0.042 | 3.368 | 1.045 - 10.853 |
| **CKD** | 1.133 | 0.462 | 6.016 | 0.014 | 3.104 | 1.256 - 7.674 |
| **Chronic liver disease** | 0.54 | 1.093 | 0.245 | 0.621 | 1.717 | 0.202 - 14.616 |
| **History of bleeding** | -0.049 | 0.627 | 0.006 | 0.938 | 0.952 | 0.279 - 3.256 |
| **Labile INR** | 0.951 | 1.13 | 0.708 | 0.4 | 2.587 | 0.282 - 23.710 |
| **Drugs** | 0.335 | 0.399 | 0.704 | 0.401 | 1.398 | 0.639 - 3.059 |
| **Alcoholism** | -0.833 | 0.742 | 1.259 | 0.262 | 0.435 | 0.101 - 1.862 |
| **Height (m)** | -3.657 | 1.747 | 4.383 | 0.036 | 0.026 | 0.001 - 0.792 |
| **Weight (kg)** | -0.018 | 0.01 | 3.218 | 0.073 | 0.982 | 0.962 - 1.002 |
| **BMI (kg/m2)** | -0.014 | 0.025 | 0.304 | 0.582 | 0.986 | 0.939 - 1.036 |
| **Creatine (mmol/L)** | 0.004 | 0.003 | 2.036 | 0.154 | 1.004 | 0.999 -1.009 |
| **Medication at baseline** | | | | | | |
| **Acetylsalicylic acid (ASA)** | -1.649 | 1.236 | 1.78 | 0.182 | 0.192 | 0.017 - 2.168 |
| **Clopidogrel** | 0.671 | 0.377 | 3.162 | 0.075 | 1.956 | 0.934 - 4.095 |
| **Ticagrelor** | -0.399 | 0.328 | 1.476 | 0.224 | 0.671 | 0.353 - 1.277 |
| **Prasugrel** | -0.286 | 0.41 | 0.487 | 0.485 | 0.751 | 0.336 - 1.678 |
| **Proton-pump inhibitors** | 1.167 | 1.029 | 1.285 | 0.257 | 3.212 | 0.427 - 24.162 |
| **Vitamin K antagonist (VKA)** | 2.367 | 1.014 | 5.45 | 0.02 | 10.667 | 1.462 - 77.819 |
| **Dabigatran (any dose)** | -18.88 | 23205 | 0 | 0.999 | 0 | 0 |
| **Dabigatrian full dose** | -18.88 | 23205 | 0 | 0.999 | 0 | 0 |
| **Rivaroxaban (any dose)** | 0.966 | 0.808 | 1.43 | 0.232 | 2.628 | 0.539 - 12.810 |
| **Rivaroxaban full dose** | -18.88 | 23205 | 0 | 0.999 | 0 | 0 |
| **Apixaban (any dose)** | 0.75 | 1.108 | 0.459 | 0.498 | 2.118 | 0.241 - 18.587 |
| **Apixaban full dose** | -18.88 | 23205 | 0 | 0.999 | 0 | 0 |
| **Any oral anticoagulation (OAC)** | 1.127 | 0.535 | 4.444 | 0.035 | 3.086 | 1.082 - 8.801 |
| **Procedural Data** | | | | | | |
| **ACS** | 18.9 | 14210.37 | 0 | 0.999 | - | - |
| **Shock** | 1.189 | 0.359 | 10.98 | 0.001 | 3.284 | 1.625 - 6.635 |
| **Cardiac Arrest** | 0.602 | 0.422 | 2.031 | 0.154 | 1.826 | 0.798 - 4.178 |
| **Femoral access** | 1.386 | 0.363 | 14.543 | <0.001 | 3.997 | 1.961 - 8.157 |
| **Procedure G2B3A used** | 1.247 | 0.34 | 13.411 | <0.001 | 3.479 | 1.785 - 6.781 |
| **TIMI flow score pre-procedure** | -0.029 | 0.168 | 0.031 | 0.861 | 0.971 | 0.698 - 1.350 |
| **DES used** | -1.547 | 0.56 | 7.636 | 0.006 | 0.213 | 0.071 - 0.638 |
| **Number of Lesions** | 0.114 | 0.165 | 0.48 | 0.488 | 1.121 | 0.811 - 1.549 |
| **Number of stents** | 0.229 | 0.142 | 2.604 | 0.107 | 1.258 | 0.952 - 1.662 |
| **LM stent** | 1.072 | 0.673 | 2.536 | 0.111 | 2.921 | 0.781 - 10.926 |
| **LAD stent** | 0.223 | 0.328 | 0.463 | 0.496 | 1.25 | 0.657 - 2.378 |
| **CX stent** | 0.076 | 0.436 | 0.03 | 0.862 | 1.079 | 0.459 - 2.535 |
| **RCA stent** | -0.229 | 0.344 | 0.444 | 0.505 | 0.795 | 0.405 - 1.561 |
| **Bypass Stent** | 1.159 | 0.834 | 2.281 | 0.131 | 3.521 | 0.687 - 18.038 |
| **Timi flow post-procedure** | -0.694 | 0.237 | 8.561 | 0.003 | 0.499 | 0.314 - 0.795 |
| **Timing chest pain-access** | -0.001 | 0.001 | 0.356 | 0.551 | 0.999 | 0.997 - 1.002 |
| **G2B3A bolus during procedure** | 1.258 | 0.341 | 13.64 | <0.001 | 3.517 | 1.804 - 6.855 |
| **G2B3A perfusion during procedure** | 0.861 | 0.331 | 6.779 | 0.009 | 2.365 | 1.237 - 4.521 |
| **Thrombo-aspiration** | -0.309 | 0.328 | 0.887 | 0.346 | 0.734 | .386 - 1.396 |
| **Use of Adenosine or Nipride** | -0.778 | 0.743 | 1.098 | 0.295 | 0.459 | .107 - 1.970 |

## **Table S3. Alternate multivariate logistic regression models for adjusted risk of bleeding based on GpIIb-IIIa strategy (conservative as reference).**

### ***Adjusted model for: Hospital, Uncontrolled HTN, HF, Any OAC***

|  | B | S.E. | Wald | Sig. | Exp(B) odds ratio | CI | |
| --- | --- | --- | --- | --- | --- | --- | --- |
|  |  |  |  |  |  | Lower | Upper |
| **Hospital** | **1.046** | **0.364** | **8.267** | **0.004** | **2.847** | **1.395** | **5.809** |
| Uncontrolled_  HTN | 1.615 | 0.613 | 6.945 | 0.008 | 5.026 | 1.513 | 16.704 |
| HF | 1.050 | 0.622 | 2.848 | 0.091 | 2.857 | 0.844 | 9.673 |
| Any_OAC | 1.020 | 0.555 | 3.376 | 0.066 | 2.773 | 0.934 | 8.232 |
| Constant | -3.156 | 0.309 | 104.220 | 0.000 | 0.043 |  |  |

### ***Adjusted model for: Hospital, Uncontrolled HTN, HF, Shock***

|  | B | S.E. | Wald | Sig. | Exp(B) odds ratio | CI | |
| --- | --- | --- | --- | --- | --- | --- | --- |
|  |  |  |  |  |  | Lower | Upper |
| **Hospital** | **0.923** | **0.369** | **6.263** | **0.012** | **2.517** | **1.222** | **5.186** |
| Uncontrolled  HTN | 1.828 | 0.619 | 8.709 | 0.003 | 6.220 | 1.848 | 20.938 |
| HF | 1.008 | 0.640 | 2.482 | 0.115 | 2.740 | 0.782 | 9.598 |
| Shock | 1.125 | 0.377 | 8.883 | 0.003 | 3.080 | 1.470 | 6.453 |
| Constant | -3.275 | 0.317 | 106.423 | 0.000 | 0.038 |  |  |

### ***Adjusted model for: Hospital, HF, Shock, CKD***

|  | B | S.E. | Wald | Sig. | Exp(B) odds ratio | CI | |
| --- | --- | --- | --- | --- | --- | --- | --- |
|  |  |  |  |  |  | Lower | Upper |
| **Hospital** | **1.029** | **0.366** | **7.902** | **0.005** | **2.800** | **1.366** | **5.739** |
| HF | 0.770 | 0.646 | 1.421 | 0.233 | 2.160 | 0.609 | 7.658 |
| Shock | 1.063 | 0.374 | 8.070 | 0.005 | 2.895 | 1.390 | 6.029 |
| Kidney | 1.108 | 0.498 | 4.945 | 0.026 | 3.030 | 1.140 | 8.048 |
| Constant | -3.321 | 0.325 | 104.475 | 0.000 | 0.036 |  |  |

### ***Adjusted model for: Hospital, Uncontrolled HTN, Femoral Access, any OAC***

|  | B | S.E. | Wald | Sig. | Exp(B) odds ratio | CI | |
| --- | --- | --- | --- | --- | --- | --- | --- |
|  |  |  |  |  |  | Lower | Upper |
| **Hospital** | **1.115** | **0.372** | **9.005** | **0.003** | **3.051** | **1.472** | **6.322** |
| Uncontrolled  HTN | 1.709 | 0.640 | 7.142 | 0.008 | 5.525 | 1.577 | 19.353 |
| Femoral Access | 1.551 | 0.386 | 16.171 | 0.000 | 4.718 | 2.215 | 10.050 |
| Any ACO | 1.248 | 0.564 | 4.890 | 0.027 | 3.483 | 1.152 | 10.529 |
| Constant | -3.494 | 0.344 | 103.456 | 0.000 | 0.030 |  |  |

### ***Adjusted Model for Hospital, HF, Shock, Femoral access***

|  | B | S.E. | Wald | Sig. | Exp(B) odds ratio | CI | |
| --- | --- | --- | --- | --- | --- | --- | --- |
|  |  |  |  |  |  | Lower | Upper |
| **Hospital** | **1.079** | **0.370** | **8.491** | **0.004** | **2.943** | **1.424** | **6.081** |
| HF | 0.831 | 0.633 | 1.725 | 0.189 | 2.296 | 0.664 | 7.935 |
| Shock | 0.710 | 0.392 | 3.278 | 0.070 | 2.034 | 0.943 | 4.386 |
| Femoral Access | 1.172 | 0.398 | 8.691 | 0.003 | 3.230 | 1.481 | 7.042 |
| Constant | -3.399 | 0.330 | 106.043 | 0.000 | 0.033 |  |  |

### ***Adjusted model for Hospital, Uncontrolled HTN. Shock, Femoral access***

|  | B | S.E. | Wald | Sig. | Exp(B) odds ratio | CI | |
| --- | --- | --- | --- | --- | --- | --- | --- |
|  |  |  |  |  |  | Lower | Upper |
| **Hospital** | **1.011** | **0.374** | **7.309** | **0.007** | **2.748** | **1.321** | **5.719** |
| Uncontrolled  HTN | 1.853 | 0.639 | 8.418 | 0.004 | 6.377 | 1.824 | 22.291 |
| Shock | 0.793 | 0.405 | 3.829 | 0.050 | 2.210 | 0.999 | 4.892 |
| Femoral Access | 1.231 | 0.405 | 9.221 | 0.002 | 3.425 | 1.547 | 7.583 |
| Constant | -3.449 | 0.334 | 106.629 | 0.000 | 0.032 |  |  |
